# Early Effect of Amyloid *β*-Peptide on Hippocampal and Serum Metabolism in Rats Studied by an Integrated Method of NMR-Based Metabolomics and ANOVA-Simultaneous Component Analysis

**DOI:** 10.1155/2017/3262495

**Published:** 2017-01-24

**Authors:** Yao Du, Hong Zheng, Huanhuan Xia, Liangcai Zhao, Wenyi Hu, Guanghui Bai, Zhihan Yan, Hongchang Gao

**Affiliations:** ^1^School of Pharmaceutical Sciences, Wenzhou Medical University, Wenzhou 325035, China; ^2^Radiology Department, The Second Affiliated Hospital, Wenzhou Medical University, Wenzhou 325027, China

## Abstract

Amyloid *β* (A*β*) deposition has been implicated in the pathogenesis of Alzheimer's disease. However, the early effect of A*β* deposition on metabolism remains unclear. In the present study, thus, we explored the metabolic changes in the hippocampus and serum during first 2 weeks of A*β*_25–35_ injection in rats by using an integrated method of NMR-based metabolomics and ANOVA-simultaneous component analysis (ASCA). Our results show that A*β*_25–35_ injection, time, and their interaction had statistically significant effects on the hippocampus and serum metabolome. Furthermore, we identified key metabolites that mainly contributed to these effects. After A*β*_25–35_ injection from 1 to 2 weeks, the levels of lactate, N-acetylaspartate, creatine, and taurine were decreased in rat hippocampus, while an increase in lactate and decreases in LDL/VLDL and glucose were observed in rat serum. Therefore, we suggest that the reduction in energy and lipid metabolism as well as an increase in anaerobic glycolysis may occur at the early stage of A*β*_25–35_ deposition.

## 1. Introduction

Alzheimer's disease (AD) has been considered as a main cause of dementia [1]. In the world, there were 24.3 million people with dementia in 2001, and this number will be raised to 42.3 million in 2020 and 81.1 million by 2040 [2]. The two main pathological features of AD are amyloid plaques and neurofibrillary tangles [1]. At present, the wildly accepted pathogenesis of AD is the amyloid cascade hypothesis, which suggests that amyloid *β* (A*β*) deposition may trigger neuronal dysfunction and death in the brain [2]. In addition, AD has been associated with a metabolic disease accompanied by impairments in glucose utilization and energy metabolism [3]. Therefore, exploring the early impact of A*β*-peptide deposited in the hippocampus on metabolism will advance understanding of the onset and development of AD.

Metabolomics is a relatively new “omics” technique that attempts to profile all low-molecular weight metabolites in biomaterials and examines their changes induced by diseases. Metabolomics has been used as a promising tool to explore the metabolic mechanisms of AD in both human patients and animal models [4]. Modern analytical platforms that have been applied for metabolomic researches mainly include mass spectrometry (MS) and nuclear magnetic resonance (NMR) spectroscopy [5]. The application of MS-based metabolomics has been reported to identify biomarkers for AD diagnosis [6–8], to monitor disease progression [9, 10], to investigate therapeutic response [11, 12], and to explore metabolic mechanisms [13–15]. Compared with mass spectrometry analysis, NMR spectroscopy as a commonly used analytical platform in metabolomics possesses several advantages, such as simple sample preparation, high reproducibility, and fast analysis. NMR-based metabolomics has been applied in exploring metabolic changes in urine, plasma, and brain of AD animal models [16, 17]. Moreover, selecting a suitable data analysis method is also of great importance for a metabolomic approach [18]. In this study, two factors, A*β*_25–35_ injection and time, were included. Thus, ANOVA-simultaneous component analysis (ASCA) was selected, since it can not only include underlying multiple factors and their interaction, but also facilitate interpreting the effects of different factors [19]. ASCA model has been successfully applied to analyze metabolomic data; for example, Yde et al. [20] used ASCA to investigate the effects of dietary, time, and their interaction on plasma metabolome of pigs and identified different absorption patterns of betaine in response to different diets. ASCA was also used to examine different sources of variations, including dose, time, and dose/time interaction of bisphenol-A exposure, on serum metabolome, and found that energy and neurotransmitter metabolism were altered by bisphenol-A [21]. Recently, the effects of dairy intake, time, and their interaction were studied on the metabolic changes in urine, blood, and feces using ASCA [22], where it was reported that high dairy consumption may result in alterations in energy, protein, and gut microbial metabolism.

A*β*_25-35_ is a peptide that exists in brain of AD patients [23]. In animals, A*β*_25-35_-administration can result in neurodegeneration [24, 25], inflammatory response [25, 26], disordered neurotransmitter metabolism [27], and impaired synaptic plasticity [4] as well as spatial learning dysfunction [4, 28]. Therefore, A*β*_25-35_ injected into the temporal cortex or hippocampus of animals has been commonly used to develop AD animal models [29]. In this study, we constructed AD rat models by injecting A*β*_25–35_ into bilateral hippocampus and examined metabolic profiles in hippocampus and serum after 1 and 2 weeks of A*β*_25–35_ injection using NMR-based metabolomics. Then, ASCA model was used to (1) analyze the effects of A*β*_25–35_ injection, time, and their interaction on metabolic changes in hippocampus and serum of rats and (2) identify metabolites related to these effects.

## 2. Materials and Methods

### 2.1. Animals

Adult male Sprague-Dawley (SD) rats (body weight = 220 ± 15 g) were purchased from the SLAC Laboratory Animal Co. Ltd. (Shanghai, China) and kept in a specific pathogen-free colony at the Laboratory Animal Center of Wenzhou Medical University (Wenzhou, China). All rats were housed in metabolic cages with regulated temperature and humidity as well as a 12/12 h light-dark cycle with lights on at 8:00 a.m. and free access to standard rat chow and tap water. The present study was conducted according to the Guide for the Care and Use of Laboratory Animals and approved by the Institutional Animal Care and Use Committee of Wenzhou Medical University.

### 2.2. Brain Stereotaxic A*β*_25–35_ Injection

Sixty SD rats were weighted and randomly divided into A*β*_25-35_ injected (AD) and control (Con) groups (30 rats for each group). After a 12 h fasting, rats were anesthetized with chloroform (3 mL/kg of body weight) and then placed on the stereotaxic apparatus to inject A*β*_25-35_ solution into bilateral hippocampus CA1. A*β*_25-35_ (Sigma Aldrich, St. Louis, MO, USA) solution was prepared by dissolving in saline at 5 mg/mL and incubating at 37°C for 7 days. Firstly, we cleaned rats' scalp by iodine solution and then incised on the midline to expose the skull. After that, two holes were drilled on both sides of the middle line (3.0 mm posterior to bregma, 2.2 mm lateral to sagittal suture, and 2.9 mm beneath the surface of brain, A/P 3.0, M/L 2.2, and D/V 2.9 [30]), followed by injecting 2 *μ*L of A*β*_25-35_ solution at the rate of 0.2 *μ*L/min. At the end of injection, the cannula was left for additional 5 min to allow sufficient diffusion of A*β*_25-35_ into the hippocampus. Rats in control group were treated with the same procedure but injected with saline.

### 2.3. Morris Water Maze (MWM) Test

The MWM test was performed to evaluate learning and memory ability in rats at 1 week after A*β*_25-35_ injection based on our previous method [31]. In brief, a circular pool with a diameter of 110 cm and a height of 30 cm was used and filled with opaque water at 22 ± 2°C. The escape platform with a diameter of 7 cm was submerged 1 cm below the surface of the water. During a 4-day training period (4 trials/day), rats were guided to reach the escape platform by the operator, if they were not able to get it within 60 s. Then, the trained rats were subjected to a 90 s probe test without the escape platform. The swimming path, mean velocity, and the number of crossings over the original platform location were recorded using a computer system.

### 2.4. Sample Collection and Preparation

The rat was sacrificed by decapitation at 1 and 2 weeks after A*β*_25-35_ injection. The hippocampus was isolated immediately, frozen using liquid nitrogen, and stored at −80°C until analysis. The hippocampus tissue was extracted according to our previous method [31]. In brief, the frozen brain tissue was weighed into an Eppendorf tube, and 4 mL/g of cold methanol and 0.85 mL/g of cold water were added into the sample tube. After homogenizing, 2 mL/g of cold chloroform and 2 mL/g of cold water were added into the mixture. Then, the mixture was homogenized using a vortex mixer, placed on ice for 15 min, and centrifuged at 10,000 ×g at 4°C for 15 min. Finally, the supernatant was carefully transferred into a new Eppendorf tube, lyophilized for 24 h, and stored at −80°C until NMR analysis. The dried extract was redissolved in 500 *μ*L of D_2_O containing 0.50 mM sodium trimethylsilyl propionate-d4 (TSP) and transferred to a 5 mm tube for NMR analysis.

Blood samples were collected from tail vein at 1 and 2 weeks after injection, centrifuged at 3,000 ×g at 4°C for 15 min to obtain serum, and stored at −80°C until NMR analysis. Prior to NMR analysis, 200 *μ*L of serum was thawed and diluted with 250 *μ*L of phosphate buffer (0.2 mM Na_2_HPO_4_/NaH_2_PO_4_, pH 7.4) to minimize pH variations and with 50 *μ*L of D_2_O for field frequency locking. Subsequently, the diluted serum was centrifuged at 12,000 ×g at 4°C for 10 min. Then, 500 *μ*L of supernatant was transferred into a 5 mm NMR tube for NMR analysis.

### 2.5. NMR-Based Metabolomic Analysis

^1^H NMR spectra were recorded using a Bruker AVANCE III 600 NMR spectrometer at 37°C. The Carr-Purcell-Meiboom-Gill (CPMG) pulse sequence was applied to reduce broad NMR signals from proteins and lipids. The main acquisition parameters were set as follows: acquisition time, 2.65 sec per scan; data points, 64 K; spectral width, 12,000 Hz; relaxation delay, 2 sec.

NMR spectra were manually phase/baseline-corrected and referenced to TSP peak at 0.00 ppm using Topspin software (v2.1 pl4, Bruker Biospin, Germany). All spectra were aligned using the “icoshift” procedure in MATLAB (R2012a, The Mathworks Inc., Natick, MA, USA) [32]. The spectral regions from 0.0 to 4.5 ppm for hippocampal sample and from 0.0 to 4.2 ppm for serum sample were subdivided and integrated to binning data with a size of 0.01 ppm for further multivariate analysis. The NMR signals were assigned using the Chenomx NMR suite 7.0 (Chenomx Inc., Edmonton, Canada) and the Human Metabolome Database [33] as well as the reported data on brain tissue [31] and serum [34].

### 2.6. ANOVA-Simultaneous Component Analysis (ASCA)

The ASCA model, which combines analysis of variance (ANOVA) and simultaneous component analysis (ACA), was used in this study. Two factors, A*β*_25-35_ injection (AD and Con) and time (1 and 2 weeks), were included in the ASCA model. Firstly, the data matrix *X* (*M* × *N*, where *M* is the number of samples and *N* is the number of variables) was separated into matrices for A*β*_25-35_ injection (*X*_*A*_) and time (*X*_*T*_) and for interaction between the two factors (*X*_*AT*_) as well as a matrix with residuals (*E*), as shown in

(1) $$\begin{array}{c} X=1m^{T}+X_{A}+X_{T}+X_{AT}+E, \end{array}$$

where 1*m* ^*T*^ is the overall means.

Then, matrices *X*_*A*_,  *X*_*T*_, and *X*_*AT*_ were decomposed into score matrices *T*_*A*_,  *T*_*T*_, and *T*_*AT*_, loading matrices *P*_*A*_,  *P*_*T*_, and *P*_*AT*_, respectively, and a residual matrix *E* as given in

(2) $$\begin{array}{c} X=1m^{T}+P_{A}^{}T_{A}+P_{T}^{}P_{T}^{}T_{T}+P_{AT}^{}T_{AT}+E, \end{array}$$

where 1*m* ^*T*^ is the overall means, *P*_*A*_  (*T*_*A*_),  *P*_*T*_  (*T*_*T*_), and *P*_*AT*_  (*T*_*AT*_) are the score (loading) matrices of A*β*_25-35_ injection, time, and their interaction, respectively, and *E* is the residual matrix.

All data were Pareto-scaled and analyzed by the ASCA model using ASCA toolbox (http://www.bdagroup.nl/Home.php) under MATLAB environment (R2012a, The Mathworks Inc., Natick, MA, USA). Moreover, the ASCA model was validated by a permutation test.

## 3. Results

### 3.1. Impaired Learning and Memory in Rats after A*β*_25–35_ Injection

**Figure 1:**
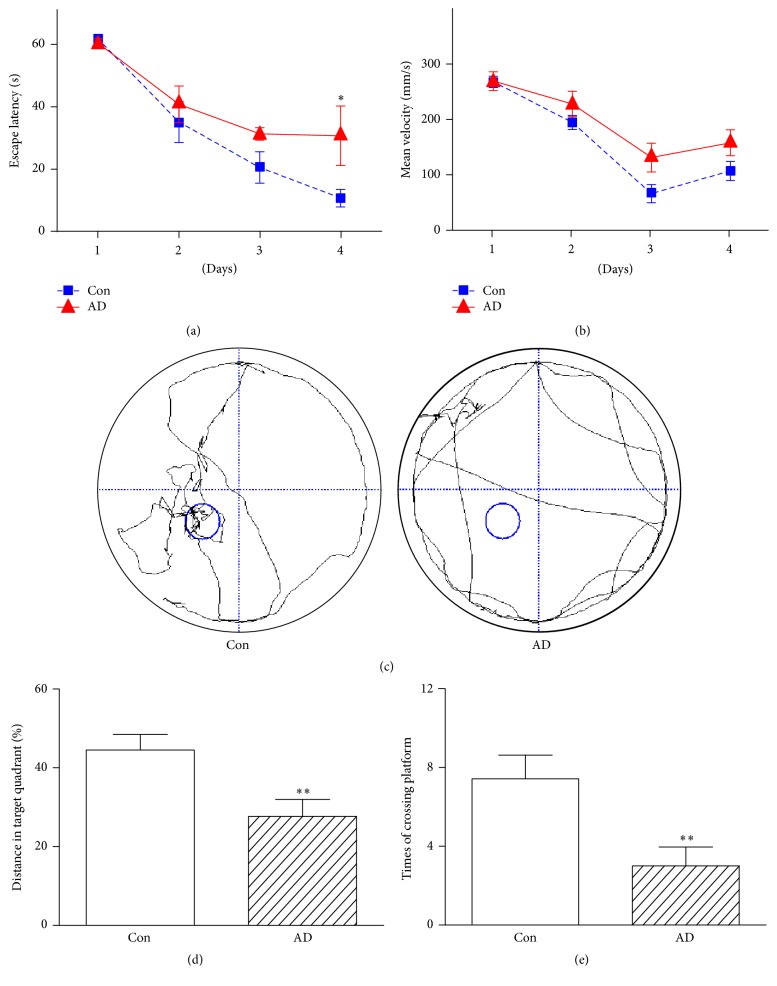
The performance of spatial learning and memory in rats after 1 week of A*β*_25-35_ injection in the Morris water maze test: (a) the escape latency and (b) the mean swimming velocity during the 4-day training period, (c) the swimming path, (d) the percentage of total swimming length in the original platform area, and (e) times of crossing the original platform in the 90 s probe test. Significant level: ^*∗*^ *P* < 0.05;  ^*∗∗*^ *P* < 0.01.

In order to assess the ability of spatial learning and memory of rats, the MWM test was concluded at 1 week after A*β*_25-35_ injection. During a 4-day training period, the escape latency in the AD rats was significantly longer than that in the Con rats on day 4 (Figure 1(a)), while no significant difference was observed in mean swimming velocity between them (Figure 1(b)).  Figure 1(c) illustrates the swimming trajectory of the AD and Con rats in the probe test of the MWM test. It can be seen that the AD rats cannot easily cross over the original platform location relative to the Con group (Figure 1(c)). Moreover, we can see that the AD rats had significantly lower percentages of total swimming length (Figure 1(d)) and time (Figure 1(e)) in the original platform area than the Con rats. Taken together, the MWM results revealed that spatial learning and memory ability was impaired in rats after A*β*_25-35_ injection.

### 3.2. ^1^H NMR Metabolite Profiles of Hippocampus and Serum in Rats

**Figure 2:**
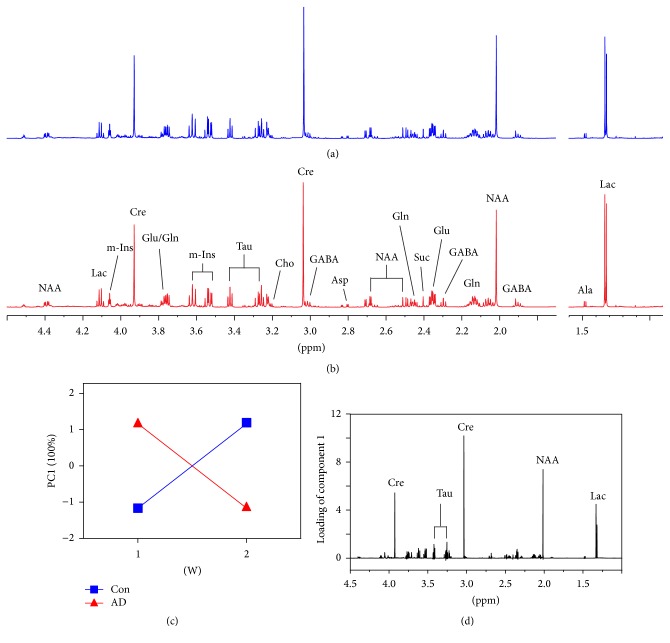
Effect of A*β*_25–35_ injection on hippocampal metabolism in rats analyzed by ASCA model: typical ^1^H NMR spectra in rat hippocampus after injection of (a) saline (b) and A*β*_25-35_ solutions; (c) ASCA score plot of the interaction effect of A*β*_25–35_ injection and time as well as (d) its corresponding loading plot. Con, the control group with saline injection; AD, the AD group with A*β*_25–35_ injection; Ala, alanine; Lac, lactate; GABA, *γ*-aminobutyric acid; NAA, N-acetylaspartate; Gln, glutamine; Glu, glutamate; Suc, succinate; Asp, aspartate; Cre, creatine; Cho, choline; Tau, taurine; m-Ins, myoinositol.

**Figure 3:**
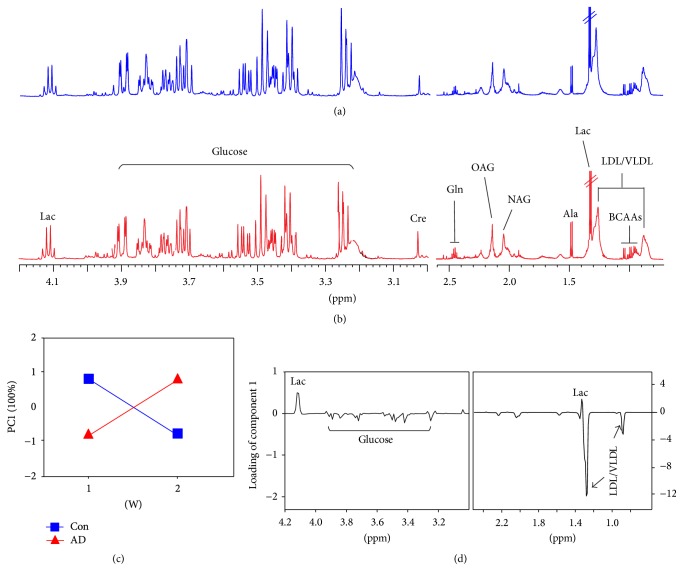
Effect of A*β*_25–35_ injection on serum metabolism in rats analyzed by ASCA model: typical ^1^H NMR spectra in rat serum after injection of (a) saline (b) and A*β*_25-35_ solutions; (c) ASCA score plot of the interaction effect of A*β*_25–35_ injection and time as well as (d) its corresponding loading plot. Con, the control group with saline injection; AD, the AD group with A*β*_25–35_ injection; LDL/VLDL, low-density lipoproteins and very low-density lipoproteins; Val, valine; Lac, lactate; Ala, alanine, NAG, N-acetylglycoprotein; OAG, O-acetylglycoprotein; Gln, glutamine; Cre, creatine; Tyr, tyrosine; His, histidine; For, formate.

Figures 2(a) and 2(b) display typical ^1^H NMR spectra of hippocampus samples obtained from the AD and Con rats, respectively. A total of 13 metabolites were identified, involving energy metabolism (Cre, creatine; Suc, succinate; Ala, alanine; Lac, lactate), neurotransmitters (Asp, aspartate; Glu, glutamate; Gln, glutamine; GABA, *γ*-aminobutyric acid; Gly, glycine), and membrane metabolism (Cho, choline; NAA, N-acetylaspartate) as well as osmoregulation (Myo, myoinositol; Tau, taurine). In addition, Figures 3(a) and 3(b) illustrate typical ^1^H NMR spectra of serum samples obtained from the AD and Con rats, respectively, and 12 metabolites were identified, such as energy metabolism (glucose; For, formate; Cre, creatine; Ala, alanine; Lac, lactate), amino acid metabolism (His, histidine; Tyr, tyrosine; Gln, glutamine; Val, valine), and lipid metabolism (LDL/VLDL, low-density and very low-density lipoproteins) as well as glycoprotein (NAG, N-acetylglycoprotein; OAG, O-acetylglycoprotein).

### 3.3. Identification of Metabolic Changes in the Hippocampus and Serum of Rats after A*β*_25–35_ Injection Using ASCA

Table 1 lists *P* values of ASCA models validated by 10,000-permutation test including the effects of A*β*_25-35_ injection, time, and their interaction (A*β*_25-35_ injection × time). We found significant effects of A*β*_25-35_ injection on the metabolite profiles in both the hippocampus (*P* = 0.0219) and serum (*P* = 0.0065). In addition, significant time effects were also observed on the hippocampus (*P* = 0.0001) and serum (*P* = 0.0001) metabolome. Most interestingly, there were significant interaction effects of A*β*_25-35_ injection and time on the hippocampus (*P* = 0.0198) and serum (*P* = 0.0001) metabolome, and the corresponding ASCA score and loading plots are illustrated in Figures 2 and 3, respectively.  Figure 2(d) shows that the levels of Lac, NAA, Cre, and Tau in the hippocampus were decreased after A*β*_25-35_ injection from 1 to 2 weeks, relative to the Con group. In the serum, we found an increase in Lac as well as a decrease in LDL/VLDL and glucose after A*β*_25-35_ injection from 1 to 2 weeks (Figure 3(d)).

## 4. Discussion

Amyloid *β*-peptide (A*β*) has been reported to induce oxidative stress in brain and thereby cause the onset and development of AD [35]. Therefore, injection of toxic A*β* into brain has been commonly used to construct AD animal model [36]. In the present study, we injected A*β*_25-35_ into hippocampal area CA1 of rats and expectedly found an impaired ability of spatial learning and memory after 1 week using the Morris water maze test. Exploring the early effect of A*β*_25-35_ deposited in the hippocampus on metabolism will achieve a better understanding of the onset and development of AD, but it has not been reported. Therefore, we examined the metabolic changes during first 2 weeks of A*β*_25-35_ injection in the hippocampus and serum of rats using an integrated method of NMR-based metabolomics and ASCA model.

### 4.1. Effect of A*β*_25–35_ Injection on Hippocampal Metabolism of Rats

The brain is vulnerable to energy metabolism deficit due to its high energy consumption [37]. In general, glucose is the main substrate for brain energy metabolism [38]. However, the astrocyte-neuron lactate shuttle (ANLS) hypothesis reported that glucose is mainly metabolized to Lac in astrocytes and then transported to neurons as the primary fuel [39]. Afterwards, Lac as a neuronal energy source was also confirmed in both* in vitro* [40] and* in vivo* [41] studies. In the present study, however, we found a reduction of Lac level in rat hippocampus after A*β*_25-35_ injection from 1 to 2 weeks compared with that in the Con rats. In addition, Cre also plays an important role in maintaining the high energetic demand for brain development and functions via the creatine kinase/phosphocreatine system [42, 43]. Like Lac, a decreased level of Cre was also observed in rat hippocampus after A*β*_25-35_ injection from 1 to 2 weeks, suggesting an insufficient supply of energy in the brain of the AD rats. A lower Cre level was also found in the hippocampus of another AD model, TgCRND8 mice [44]. Growing evidences have supported the concept that AD is a metabolic disease with impaired energy metabolism [45–47]. Moreover, González-Domínguez et al. [48] have also found that impaired energy metabolism is a key cause in pathogenesis of Alzheimer. Therefore, a reduction in energy metabolism in the hippocampus may be implicated in the onset and development of AD.

NAA has been regarded as a marker of neuronal density and integrity, since it is exclusively synthesized in the mitochondria of neurons [49, 50]. We found that NAA level was reduced in rat hippocampus after A*β*_25-35_ injection relative to the Con group from 1 to 2 weeks, indicating that hippocampal neurons may be damaged during the progress of AD in rats. Previous findings have also shown that the level of NAA was decreased in neurodegenerative diseases, such as Alzheimer's disease and Huntington's disease [51–53]. Lalande et al. [54] also found a reduction of NAA in Tg2576 mice at one month of age relative to the age-matched wild-type mice. Tau is an important indicator of astrocyte as well as a regulator of osmotic pressure [55, 56]. Meanwhile, it also acts as an antioxidant in neuroprotection [57, 58]. In this study, compared with the Con group, a decrease in hippocampal Tau level in rats after A*β*_25-35_ injection from 1 to 2 weeks may reflect the reduction of astrocyte activity and antioxygenation in the hippocampus. A similar finding was also obtained in the hippocampus of TgCRND8 mice [44]. Taken together, decreased levels of NAA and Tau may indicate that astrocytes and neurons in the hippocampus were damaged during AD development in rats.

### 4.2. Effect of A*β*_25–35_ Injection on Serum Metabolism of Rats

In this study, VLDL/LDL, glucose, and lactate in serum were identified as the main metabolites induced by A*β*_25-35_ injection using an integrated analytical method of NMR-based metabolomics and ASCA model. The initiation and acceleration of AD pathology have been closely associated with the downregulation of lipid metabolism [59, 60]. In the current study, we found that the serum level of lipoprotein, VLDL/LDL, was reduced after A*β*_25-35_ injection from 1 to 2 weeks as compared with the Con group. Lipoprotein particles are lipid transporters in blood, so our finding may indicate a reduction of lipid metabolism in the AD rats. De La Monte et al. [45] found that lipid derivatives including phospholipids and sphingomyelins were significantly decreased in APP/PS1 mice. Glucose is the predominant energy substrate that can be oxidized to CO_2_ and H_2_O through tricarboxylic acid (TCA) cycle or transformed into lactate by anaerobic glycolysis. In this study, an increase in lactate level and a decrease in glucose level were found in the serum after A*β*_25-35_ injection from 1 to 2 weeks, indicating that anaerobic glycolysis was upregulated during AD development. Using ^1^H NMR-based metabolomic approach, similar results were also observed in APP/PS1 [7] and senescence-accelerated mouse prone 8 (SAMP8) [46] mice. In addition, compared with the wild-type mice, De La Monte et al. [45] found reduced glucose and increased lactate in serum of the APP/PS1 mice using a MS-based metabolomic approach. Therefore, serum metabolomics analysis reveals that a decrease in lipid metabolism and an increase in anaerobic glycolysis may be associated with the development of AD in rats.

In conclusion, the early effect of A*β*_25-35_ deposition on hippocampal and serum metabolism was explored by an integrated analytical method of NMR-based metabolomics and ASCA model. Our results revealed that a series of metabolic disorders occurred at the early stage of A*β*_25-35_ deposition, including reduction in energy metabolism in the hippocampus as well as a decrease in lipid metabolism and an increase in anaerobic glycolysis in the serum. However, several limitations in the present study should be considered: (1) these findings should be confirmed by the use of multidose A*β*_25-35_ and other animal models; (2) only a few metabolites induced by A*β*_25-35_ injection were identified, so we suggest using a multianalytical platform for drawing a more detailed metabolic pathway; (3) measuring key proteins/enzymes in metabolic pathways will advance understanding of the metabolic mechanism underlying the onset and development of AD.

## Figures and Tables

**Table 1:** *P* values of ASCA models by 10,000-permutation test.

Samples	A*β*_25–35_	Time	A*β*_25–35_ × time
Hippocampus	0.0219	0.0001	0.0198
Serum	0.0065	0.0001	0.0001
